# Willingness, preferences, barriers, and facilitators of a multimodal supportive care intervention including exercise, nutritional and psychological approach in patients with cancer: a cross-sectional study

**DOI:** 10.1007/s00432-022-04232-6

**Published:** 2022-08-09

**Authors:** Alice Avancini, Ilaria Trestini, Daniela Tregnago, Lorenzo Belluomini, Marco Sposito, Jessica Insolda, Federico Schena, Michele Milella, Sara Pilotto

**Affiliations:** 1 Section of Oncology, Department of Medicine, University of Verona School of Medicine and Verona University Hospital Trust, Verona, Italy; 2 Department of Neurosciences, Biomedicine and Movement Sciences, University of Verona, P.le L.A. Scuro 10, 37134 Verona, Italy; 3 Dietetics Services-Medical Direction, University Hospital of Verona, Verona, Italy

**Keywords:** Cancer, Exercise level, Nutritional risk, Psychological distress, Multimodal intervention

## Abstract

**Purpose:**

Supportive care, including exercise, nutritional and psychological support, is becoming increasingly important in cancer given their impact on ‘patients’ quality and quantity of life. The purpose of this study was to explore willingness, preferences barriers and facilitators for a multimodal intervention in patients with cancer.

**Methods:**

An anonymous questionnaire was proposed on randomly selected days to the patients visiting the cancer outpatients’ facilities at the Oncology Unit of the University Hospital of Verona. The questionnaire investigated willingness, preferences, barriers, and facilitators associated with participation in a multimodal program designed for patients with cancer. Exercise level was estimated using two open questions, nutritional risk was identified using the Nutritional Risk Screening 2002, while distress was evaluated with the Distress Thermometer.

**Results:**

Based on 324 participants, 65% were interested in starting a multimodal intervention. Patients declared to prefer to receive instructions from dedicated experts, with a face-to-face approach, and during the anticancer treatment. Treatment-related side effects were the major obstacles for a multimodal program, while the availability of a specialized staff as exercise kinesiologists, dietitians, and psycho-oncologists was found to be an important facilitator for increasing ‘patients’ participation.

**Conclusion:**

Patients patients with cancer are interested in participating in a multimodal supportive care program specifically designed for them. Information from this study may help to design a tailored multimodal intervention for patients with cancer.

**Supplementary Information:**

The online version contains supplementary material available at 10.1007/s00432-022-04232-6.

## Introduction

Whereas cancer incidence increased over the years (one out of three men and one out of four women are expected to develop cancer in their lifetime), cancer mortality decreased in many countries, leading to a high prevalence of people living with cancer (Fitzmaurice et al. 2017). Nevertheless, malignancy and its treatments are often related to several side effects, potentially impairing patients’ quality of life for years even after the therapy conclusion (Devlin et al. 2017). In this sense, patients with cancer may experience a range of different symptoms and adverse events affecting their physical and psychological well-being, such as functional deconditioning, nausea, vomiting, loss of muscle mass, anxiety, and depression (Devlin et al. 2017). This scenario emphasizes the need for supportive care programs aimed to help patients in recovering from the cancer pathway. Indeed, supportive care, defined as “the prevention and management of the adverse effects of cancer and its treatment” (Berman et al. 2020), is an essential tool able to manage physical and psychological symptoms and enhance the rehabilitation and survivorship of patients. Among them, exercise, nutrition, and psychological support are important for patients with cancer. Individually, these interventions have been shown to bring a series of benefits. Exercise can increase patients' physical fitness, such as cardiorespiratory fitness, muscle mass, and strength, which are prognostic factors in patients with cancer (Campbell et al. 2019). Moreover, exercise may ameliorate adverse events of cancer and its treatments, manage cancer-related fatigue, anemia, and psychological impairments, improving peripheral neuropathy and quality of life (Campbell et al. 2019). Similarly, a nutritional screening may help to individuate, and consequently manage, malnourished patients or those at risk of malnutrition (Rock et al. 2022; Trestini et al. 2020). Nutritional intervention may facilitate maintaining an adequate nutritional intake, controlling body composition, and nutritional impact symptoms, such as nausea, vomiting, and appetite loss, to prevent loss of muscle mass and nutrient inadequacies (Caccialanza et al. 2022; Rock et al. 2022; Trestini et al. 2021a, b; Trestini et al. 2021a, b). A cancer diagnosis carries an important psychological burden for patients and their caregivers. Symptoms of anxiety, depression, fear of death, and/or recurrence frequently occur in patients with cancer. Psychological interventions, e.g., cognitive–behavioral therapy, address various psychological and social aspects that may alleviate the emotional outcomes, improving patients' quality of life (Liu et al. 2019; Travier et al. 2015).

Exercise, nutrition, and psychological aspects are strictly related, and it is reasonable to speculate that a supportive care multimodal approach could grow the benefits, as supported by previous research (Avancini et al. 2021). An 18 week exercise program including cognitive–behavioral principles of social cognitive theory found positive effects on fatigue and physical fitness levels in patients with breast cancer receiving chemotherapy (Travier et al. 2015). Similarly, a two-week prehabilitation program incorporating exercise, nutritional counseling, and psychological guidance has been shown to be effective in increasing perioperative functional capacity in patients with lung cancer undergoing thoracoscopic lobectomy (Liu et al. 2019). Nevertheless, investigations combining exercise, nutritional and psychological approaches are still few, and more research on the benefit of multimodal interventions is necessary. However, participating in a supportive care multimodal program requires a time effort from patients, making high the risk of non-compliance.

Understanding patients’ willingness, preferences, barriers, and needs may permit to develop an optimal and tailored multimodal intervention. Although the factors influencing the single lifestyle intervention in a population with cancer are investigated, studies exploring the feasibility and the willingness of patients to join a multimodal intervention are lacking. To fill this gap, this research aims to: (i) establish the willingness of patients with cancer to participate in a multimodal supportive care intervention, including exercise, nutritional and psychological approaches, (ii) analyze the patients' characteristics associated with their willingness to participate in a multimodal supportive care intervention, (iii) describe patients’ preferences about exercise, nutritional and psychological interventions, (iv) examine the perceived barriers and facilitators affecting patients adherence to the multimodal intervention.

## Methods

### Study design and participants

A cross-sectional survey was utilized. Between February 2020 and May 2021, an anonymous questionnaire was delivered to the patients visiting the cancer outpatients' facilities at the Oncology Unit of the University Hospital of Verona. Patients' eligibility criteria were: 18 years or older, a cancer diagnosis, and ability to understand Italian. The study staff distributed the questionnaires on randomly selected days. Patients were approached face-to-face, informed about the study, and asked whether they would be willing to complete the questionnaire. If interested, patients signed the informed consent and received a copy of the questionnaire to return directly. A duplicate check was done, looking for duplicates by date of birth, sex, education, and marital status. Approval of the Ethics Committee for Clinical Trials (Prot. No. 48647) was obtained. The study protocol adhered to Good Clinical Practice principles and the procedures were conducted following the last revision of the declaration of Helsinki as well as the declaration of Oviedo. The current report is compliant with the Strengthening the Reporting of Observational Studies in Epidemiology (STROBE) guidelines (Cuschieri 2019).

### Questionnaire description

A self-administered survey questionnaire was developed to collect preferences, barriers, and facilitators associated with a multimodal intervention. A pilot version of the questionnaire was created using questions derived from previous studies (Arthur et al. 2016; Avancini et al. 2020a; Weller et al. 2019), and made available to experts, including kinesiologists, oncologists, and psycho-oncologists, to make an informal peer review and develop the current version.

The questionnaire comprised 30 items and took approximately 30 min to complete. The survey included five parts: (i) General characteristics; (ii) Distress, exercise level, and risk of malnutrition; (iii) Multimodal intervention preferences; (iv) Barriers and facilitators associated with a multimodal intervention; (v) Cancer diagnosis and treatment.

#### General characteristics

The following demographic, and socio-economic factors were self-reported: birth date (day, month, year), sex (male/female), educational level (elementary—up to age 10–11 years/secondary—up to 14 years/secondary—up to 18–19 years/college–university/postgraduate), marital status (single/married/divorced/widowed), occupational status (retired/ homemaker/part-time employed/ full-time employed), perceived economic adequacy (inadequate/ barely adequate/adequate/ more than adequate). Weight in kilograms and height in meters were collected to obtain body mass index (BMI). BMI was calculated dividing the weight by the squared height, and categorizing as: underweight (BMI < 18.5 kg/m^2^), normal weight (BMI 18.5–24.9 kg/m^2^), overweight (BMI 25.0–29.9 kg/m^2^) and obese (BMI > 29.9 kg/m^2^) (“Physical status: the use and interpretation of anthropometry. Report of a WHO Expert Committee” 1995).

#### Distress, exercise level, and risk of malnutrition

Distress was assessed using the National Comprehensive Cancer Network (NCCN) Distress screening tool (Riba et al. 2019). Through a single item, representing an 11 points visual/Likert scale, from 0 (no distress) to 10 (extreme distress), the patient was asked to rate her/his level of distress experienced over the past week. Distress is classified as mild with a cutoff point < 4, whereas a score > 3 requires further screening (Riba et al. 2019). Exercise level was assessed with the two open-ended questions, adapted by Schmitz et al. (Schmitz et al. 2019): “How many days per week and times per session do you perform an aerobic activity at moderate intensity (where the heart beats faster and the breathing harder than normal, e.g., walking, cycling, running, swimming)? and “How many days per week do you perform exercise to increase muscle strength (e.g., lifting weights, bodyweight exercise, climbing)? According to the American College of Sports Medicine guidelines for patients with cancer, patients were defined as meeting the guidelines if they engaged in at least 90 min/week of aerobic exercise and performed strength activities at least two times/week (Campbell et al. 2019). The Nutritional Risk Screening 2002 (NRS-2002) was used to identify the nutritional risk. Malnutrition risk identification was not directly captured, but the questionnaire included nutrition-related questions, including severity of the disease, weight loss in the past one, two, and three months, perceived impairments in general condition, and the reduction of food intake in the preceding week, that allowed us to estimate its prevalence. NRS-2002 is evaluated through three components: nutritional status (0–3 points), the severity of disease (0–3 points), and age (0–1 points). The total NRS-2002 score ranges from 0 to 7, and patients with a score of < 3 and ≥ 3 are classified as “no nutritional risk” and “at nutritional risk”, respectively (Kondrup et al. 2003).

#### Multimodal intervention preferences

Preferences were explored using closed-item questions adapted from prior investigations. The first question concerned the patients’ willingness to participate in a multimodal supportive care intervention including exercise, nutritional counseling and psychological support specifically designed for patients with cancer (yes/no/maybe).

For each intervention, i.e., exercise, nutrition and psychological support, were asked patient ‘preferences concerning: who would give them instructions (oncologist/nurse/dietitian/kinesiologist/psycho-oncologist/another patient with cancer/other); how to receive instructions (face to face/over the internet/television/radio/brochure-pamphlet/other); where (at hospital/a community center outside the hospital/other); when (before treatment/during treatment/after treatment/other).

#### Barriers and facilitators associated with a multimodal intervention

A list of potential barriers and facilitators associated with participation in a multimodal intervention was provided. Patients may select up to three barriers and up to three facilitators.

#### Cancer diagnosis and treatment

Self-reported medical variables included: cancer site (breast/lung/colorectal/upper gastrointestinal/head-neck/gynecological/urogenital/melanoma/other), disease status (early/advanced/metastatic/in remission-cured/unknown), date of diagnosis (month/year), type of treatment (surgery/chemotherapy/radiotherapy/hormone therapy/other), and current treatment status (about to start/ongoing/completed/not known).

### Statistical analysis

Descriptive analyses were utilized to summarize the response to survey questions. Categorical data were presented as frequencies and percentages. Logistic regression models were applied to identify patients’ characteristics (sex, age, education, body mass index, exercise level, psychological distress, risk of malnutrition, marital status, occupational status, perceived income adequacy, tumor site, disease status, cancer treatment, treatment status, and time from diagnosis) associated with their willingness to participate in a multimodal supportive care program. Additionally, logistic regression models were applied to explore patients’ characteristics associated with exercise level, risk of malnutrition and distress (Supplementary Material 1). SPSS version 28.0 software was utilized to analyze data. The significance level was set at 0.05, whereas all statistical tests were two-sided.

## Results

Between February 2020 and May 2021, a total of 623 were approached, and among them, 324 agreed to participate in the survey (52% response rate).

### General characteristics

The demographic and medical characteristics of the survey respondents are listed in Table 1. Overall, 57% had less than 65 years, 53% were female, 61% had a higher education (at least up to age 18–19 years), and 70% were married. Upper gastro-intestine (44%) and breast (17%) were the most frequent cancer site, and about 77% were on active anticancer treatment.

**Table 1:** General characteristics of the survey’s participants^1^

	No	%
Age (years) (*n* = 324)		
< 65	184	57
≥ 65	140	43
Sex (*n* = 324)		
Female	171	53
Male	183	47
Education (*n* = 324)		
Elementary (up to 10–11 years)	27	8
Secondary (up to 14 years)	101	31
Secondary (up to 18–19 years)	120	37
College/University	67	21
Postgraduate	9	3
Body Mass Index^2^ (*n* = 324)		
Underweight	12	4
Normal weight	134	41
Overweight	133	41
Obese	45	14
Marital status (*n* = 324)		
Single	35	11
Married	228	70
Divorced	33	10
Widowed	28	9
Occupational status (*n* = 324)		
Retired	154	48
Homemaker	47	16
Part-time employed	24	7
Full-time employed	93	29
Other	0	0
Perceived income adequacy^3^ (*n* = 324)		
Inadequate	14	4
Barely adequate	59	18
Adequate	159	49
More than adequate	92	28
Exercise level (*n* = 314)		
Meeting aerobic guidelines		
Yes	94	30
No	220	70
Meeting strength guidelines		
Yes	31	10
No	283	90
Meeting exercise guidelines		
Yes	11	4
No	303	96
Distress level (*n* = 306)		
Mild	129	42
Clinically elevate	177	58
Risk of malnutrition (*n *= 320)		
Yes	97	30
No	223	70
Tumor site (*n* = 324)		
Breast	55	17
Lung	18	6
Colorectum	20	6
Head/neck	19	6
Upper gastro-intestine	143	44
Gynecological	4	1
Urogenital	24	7
Melanoma	22	7
Other	19	6
Disease status (*n* = 324)		
Unknown	59	18
In remission/cured	67	21
Early	44	14
Advanced	72	22
Metastatic	82	25
Treatments^4^ (*n* = 324)		
Surgery	157	48
Chemotherapy	221	68
Radiation therapy	73	23
Hormone therapy	22	7
Other	56	17
Treatment status (*n* = 324)		
About to start	16	5
Ongoing	234	77
Completed	43	14
Unknown	12	4
Time from diagnosis (*n* = 324)		
≤ 30 months	186	57
≥ 30 months	138	43

^1^Participants of survey study conducted in Verona, Italy, from February 2020 to May 2021

^2^Body mass index categories are those of the World Health Organization]

^3^Perceived income adequacy assessed by the question: does your monthly income cover your monthly expenditure*?*

^4^Treatments, which may be completed or in course, and are not mutually exclusive

Overall, 30% and 10% of patients reported following the amount of exercise suggested for aerobic and strength activities, respectively. When the two types of exercise were merged, only 4% of survey participants resulted to follow the ACSM guidelines. Whereas the aerobic exercise levels were similar in males and females and through the age, strength training was higher in male patients with < 65 years (Supplementary material 1). The Distress Thermometer found clinically relevant levels of distress in 58% of survey participants, with percentages slightly higher in female patients. A total of 30% of patients were at risk of malnutrition. Older patients, both male and female were more frequently at high risk of malnutrition compared to < 65 years. Logistic regression models found several patients’ characteristics associated with exercise level, risk of malnutrition and distress (Supplementary Material 1).

### Willingness and preferences for a multimodal supportive care intervention

**Fig. 1:**
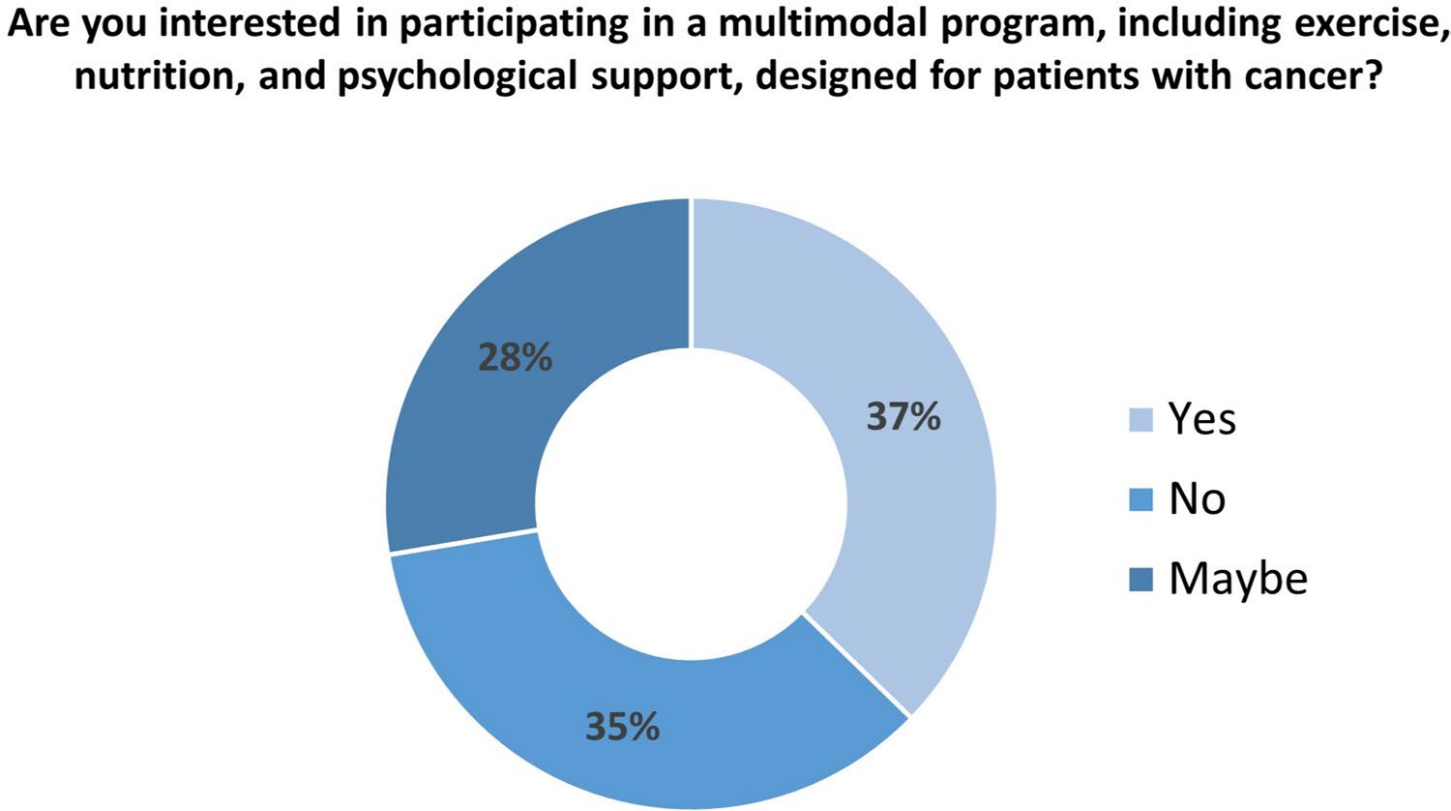
Willingness to start a multimodal intervention including exercise, nutritional counseling, and psychological support

The willingness to participate to a multimodal supportive care program is shown in Fig. 1. A total of 65% of the survey participants were interested (i.e., yes, or maybe) in participating in a multimodal program, including exercise, nutrition, and psychological support, specifically designed for patients with cancer. Compared to patients having < 65 years, older subjects were less willing to participate in a multimodal intervention (OR = 0.42, 95% CI = 0.26–0.69). Patients who were single were more likely to join the multimodal intervention (OR = 2.24, 95% CI = 1.08–4.63) than those married. Patients who self-defined their cancer stage as "early were less interested in participating in an intervention, including exercise, nutrition, and psychological support (OR = 0.34, 95% CI = 0.14–0.86). Logistic regression is displayed in Supplementary material 1.

Survey ‘respondents’ preferences are listed in Table 2. Patients preferred to receive instructions from the reference experts for each area: a kinesiologist (48%) for exercise, a dietitian (61%) for nutrition, and a psycho-oncologist (61%) for psychological support. For each intervention, the preferred way to receive information was through a face-to-face approach (66% for exercise, 69% for nutrition, and 72% for psychological support), at the hospital (78% for exercise, 87% for nutrition, and 88% for psychological support), and during anticancer treatment (44% for exercise, 47% for nutrition, and 51% for psychological support).

**Table 2:** Preferences in patient with cancer for exercise, nutrition, and psychological aspect^1^

Preference as expressed by answers to questions	Exercise		Nutrition		Psychology	
	*N*	%	*N*	%	*N*	%
Who would you prefer to receive instructions from?	(*n* = 304)		(*n* = 312)		(*n* = 305)	
Oncologist	125	41	112	36	102	33
Nurse	2	1	1	0	2	1
Kinesiologist	145	48	4	1	6	2
Nutritionist	14	5	191	61	2	1
Psychologist	2	1	3	1	186	61
Another patient cancer	0	0	0	0	0	0
No preference	0	0	0	0	0	0
Other	16	5	1	0	7	2
How would you prefer to receive instructions?	(*n* = 300)		(*n* = 305)		(*n* = 301)	
Face to face	197	66	209	69	218	72
Television	8	3	7	2	6	2
Radio	1	0	1	0	1	0
Leaflet/pamphlet	23	8	18	6	13	4
Over the internet	60	20	61	20	51	17
No preference	0	0	0	0	0	0
Other	11	4	9	3	12	4
Where would you prefer to receive instructions?	(*n* = 310)		(*n* = 306)		(*n* = 306)	
At the hospital	240	78	271	87	264	88
At a center outside the hospital	57	19	33	11	35	11
Other	9	3	6	2	7	2
When would you prefer to receive instructions?	(*n* = 300)		(*n* = 294)		(*n* = 292)	
Before treatment	85	29	89	30	74	25
During treatment	130	44	142	47	148	51
After treatment	34	12	26	9	27	9
Other	45	15	43	14	43	15

^1^Participants of survey study conducted in Verona, Italy, from February 2020 to May 2021

### Barriers and facilitators to a multimodal intervention

**Fig. 2:**
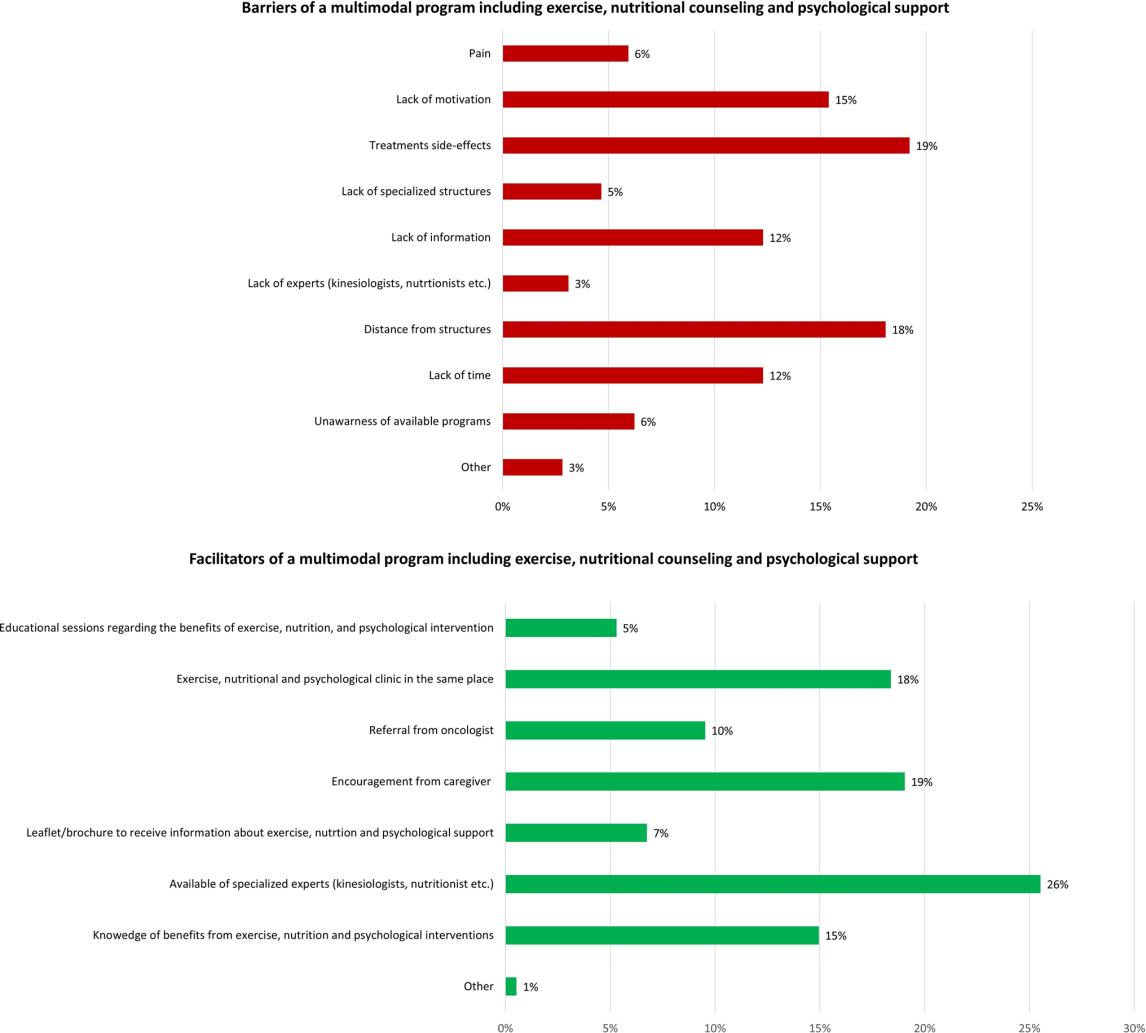
Barriers and facilitators associated with a multimodal intervention

Figure 2 reported the barriers and facilitators of a multimodal program individuated by patients. The most commonly reported barriers potentially hindering participation in a multimodal supportive care intervention were: treatment-related side effects, distance from the structures, and lack of motivation. On the contrary, most commonly reported features facilitating the involvement in a program, including exercise, nutrition, and psychological support, were: availability of specialized experts, encouragement from caregivers, and having the exercise, nutritional and psychological clinics in the same place.

## Discussion

The present study is the first to explore willingness, preferences, barriers, and facilitators among patients with cancer to participate in a multimodal supportive care program including exercise, nutritional counseling, and psychological support.

We found that roughly 65% of the survey participants were willing to start a multimodal program, including exercise, nutrition, and psychological support, designed for patients with cancer. This finding is particularly crucial especially if considered that 30% of patients were at nutritional risk, 58% reported clinically relevant levels of distress, and 96% did not meet the current exercise guidelines for cancer patients, and the possible consequences in terms of quality and quantity of life of such impaired levels (Gerritsen and Vincent 2016; Hamer et al. 2009; Patel et al. 2019; Zhang et al. 2019). Prior investigations reported that patients with cancer usually express interest in health-related behaviors intervention, such as exercise (Avancini et al. 2020a), nutritional counseling (Green et al. 2014; Keaver et al. 2022), and psychological support (Arch et al. 2018). Similarly, a study exploring the preferences related to physical activity and diet interventions in pancreatic cancer survivors found that 69% of study participants were interested in participating a combined lifestyle intervention (Arthur et al. 2016). Our results support the desire of the patients with cancer to be proactive in their disease journey and their wish for a multimodal supportive care service. Moreover, this finding is crucial, especially in view of the multidisciplinary approach delivery and from a clinical point of view (Avancini et al. 2022). In this sense, different societies support the integration of supportive care alongside the cancer continuum. For instance, prehabilitation, i.e., interventions, such as exercise, smoking cessation, nutrition, and psychological support, performed between the time of cancer diagnosis and the beginning of the acute treatment, is becoming more and more relevant and a standard of care so much that different guidelines insert it in their recommendations (Low et al. 2019; Melloul et al. 2020; Nelson et al. 2019). On the other hand, the multimodal intervention has been suggested as a possible treatment for a complex syndrome like cancer-related cachexia. This multifactorial syndrome, characterized predominantly by loss of skeletal muscle mass, not fully reversed by nutritional support, and leading to a progressive functional impairment, is still orphan of effective treatments (Avancini et al. 2021). Whereas single interventions failed to gain benefits, multimodal management may be the best strategy, given its multifactorial nature (Avancini et al. 2021).

Logistic regression revealed that younger patients and those who were single were more interested in participating in a multimodal supportive care intervention. These results may be explained by the fact that aging is associated with comorbidities and growing difficulties. Prior researches on exercise found mixed results. On one side, some studies reported that more than half of older patients with cancer were interested in participating in a physical activity program (Cheung et al. 2021) (Fournier et al. 2022). On the contrary, a cross-sectional study on ovarian cancer survivors found that the interest in physical activity diminished in among participants who were aged ≥ 60 years (Stevinson et al. 2009), and Morielli and colleagues reported that age was associated with a low exercise adherence in patients with rectal cancer (Morielli et al. 2018). Regarding medical variables, patients who defined their cancer at “early” stage were less willing to participate in a multimodal supportive care program. Despite, to our knowledge, no prior studies reported a similar finding; this could intuitively explain by the fact that patients with an early stage of disease might feel less in danger, while individuals with an advanced/metastatic cancer, being aware of the severity of their disease, would try everything to feel better (Avancini et al. 2020a; Wong et al. 2018).

Regarding the preferred source for receiving instructions about each intervention, patients preferred to receive information from a dedicated expert (i.e., kinesiologist, dietitian, psycho-oncologist). These results are in line with previous studies (Gjerset et al. 2011; Nicole Culos-Reed et al. 2017), and with recommendations of international societies (Arends et al. 2017; Campbell et al. 2019). Nevertheless, a relevant percentage of patients, ranging from 33 to 41%, have indicated the oncologist as the preferred person to deliver instructions. Similarly, our previous work focusing on exercise identified the oncologist as the preferred person to deliver information (Avancini et al. 2020a). These results may be explained by the fact that the patients recognize the specificity of each approach and thus the need for specialized staff. Nevertheless, patients put their trust in their oncologists during the cancer journey, and therefore some of them may feel more reassured to receive instruction from their voice.

Most patients reported that they preferred to receive exercise and nutritional and psychological instructions face-to-face. Whereas face-to-face counseling is the most preferred counseling modality in several previous investigations (Avancini et al. 2020a; Wong et al. 2018), we found that a quarter of patients have indicated the internet as the preferred source of information. Information over the internet may have the great advantages of reaching a large number of individuals, increasing patients’ knowledge and engagement in health decision-making strategies. On the other hand, web-based informations are difficult to regulate, and often the quality control is a challenge, in which the risk of incurring misleading information becomes high, hitting more individuals in a vulnerable position, such as patients with cancer. In this sense, providing reliable websites developed by reputable institutions, such as universities or hospitals, may overcome this problem and offer evidence-based information.

The hospital was identified as the preferred place to receive information. Moreover, patients wished to receive instruction about the multimodal intervention before the beginning of therapies or during anticancer treatment. Although the time variance in the preference of lifestyle program start has been reported in the literature (Wong et al. 2018), our results are encouraging because such interventions (or the monitoring through appropriate screening tools), should be early and regularly administered (Ravasco 2019) to prevent or manage possible impairments.

Treatment side effects, distance from facilities, and lack of motivation were reported as the major barriers. These results of mixed disease specific and general obstacles to multimodal intervention mirror the current literature (Arthur et al. 2016; Avancini et al. 2020a; Clifford et al. 2018; Keaver et al. 2022). Although treatment-related adverse events, such as fatigue, and lymphoedema, are frequently reported as potential barriers in lifestyle interventions (Arthur et al. 2016), it is interesting to highlight that many of these side effects can effectively be managed through supportive care intervention. In this sense, increasing patients’ knowledge and awareness about the benefits of supportive care intervention may be a useful strategy to increase patients’ compliance. Distance from facilities and lack of motivation may be easily overcome, including tailored programs with different modality options (e.g., face-to-face approach or using telehealth), and incorporating motivational approach (e.g., goal setting, self-monitoring, action planning) to behavior change.

On the other, the availability of specialized experts, having exercise, nutrition, and psychological clinic in the same facilities, and social support were identified as facilitators for the participation in a multimodal supportive care program. Prior investigations support the experts’ supervision as facilitators to participate in a lifestyle program (Avancini et al. 2020a, b, c; Keaver et al. 2022), and may suggest that patients desire tailored intervention based on their needs. Social support may play a role in the intervention compliance, supporting patients ‘motivation and enhancing emotional well-being (Fong et al. 2017).

The present work has some limitations that should be noted. Information was self-reported and therefore open to different sources of bias. Recall bias may be a possible source of error. To minimize this issue, we have adopted the short version of the questionnaire investigating exercise, nutritional risk, and distress, asking for recent information (e.g., in the previous week). Moreover, the questionnaire did not collect information regarding participants’ diet, and that limit its ability to explore associations with other possible determinants of willingness to participate in a multimodal lifestyle program. The social desirability bias may be less likely because the questionnaire was filled and returned anonymously.

However, it cannot be excluded that patients who decided to participate in this study may be individuals more interested in the supportive care. To reduce this potential bias, the questionnaire was proposed to all patients on randomly selected days. The study participants were sampled to be representative of those attending the Verona oncology clinic, and not the full total of the patients, making our results little generalizable.

In conclusion, this study highlights that patients desire to participate in a multimodal supportive care intervention, including exercise, nutritional and psychological support, specifically designed for individuals with cancer. Patients prefer to receive instruction in the hospital, from dedicated experts, and with a face-to-face approach. Although different barriers to multimodal supportive care intervention have been identified, several facilitators may promote patient compliance.

Overall, these results support that a multimodal supportive care intervention is feasible and desired by patients with cancer, and represent the first step toward the development of a tailored program.

## Supplementary Information

Below is the link to the electronic supplementary material.

Supplementary file1 (DOCX 56 KB)
