# Supplementary material for: Willingness, preferences, barriers, and facilitators of a multimodal supportive care intervention including exercise, nutritional and psychological approach in patients with cancer: a cross-sectional study
Source: J Cancer Res Clin Oncol. 2022 Aug 9;149(7):3435–45. doi: 10.1007/s00432-022-04232-6 (PMC10314831; doi:10.1007/s00432-022-04232-6)
Supplement: Supplementary file 1 — Supplementary file1 (DOCX 56 KB) [file 432_2022_4232_MOESM1_ESM.docx]

**Table 1.** Characteristics of exercise level, psychological distress, and risk of malnutrition in patients with cancer^1^ by age and sex.

|  | **All patients** | | **Age <65years** | | | | **Age ≥65years** | | | |
| --- | --- | --- | --- | --- | --- | --- | --- | --- | --- | --- |
|  | (n=324)^2^ | | **Men**  (n=71)^2^ | | **Women**  (n=113)^2^ | | **Men**  (n=81)^2^ | | **Women**  (n=59)^2^ | |
| **Exercise level**^3^ | N | % | N | % | N | % | N | % | N | % |
| *Meeting aerobic guidelines* |  |  |  |  |  |  |  |  |  |  |
| Yes | 94 | 30 | 24 | 35 | 30 | 27 | 25 | 32 | 15 | 27 |
| No | 220 | 70 | 45 | 65 | 82 | 73 | 52 | 68 | 41 | 73 |
| *Meeting strength guidelines* |  |  |  |  |  |  |  |  |  |  |
| Yes | 31 | 10 | 12 | 18 | 10 | 9 | 8 | 10 | 1 | 2 |
| No | 283 | 90 | 56 | 82 | 102 | 91 | 70 | 90 | 55 | 98 |
| *Meeting exercise guidelines* |  |  |  |  |  |  |  |  |  |  |
| Yes | 11 | 4 | 6 | 9 | 4 | 4 | 34 | 5 | 1 | 2 |
| No | 303 | 96 | 62 | 91 | 108 | 96 | 74 | 95 | 55 | 98 |
| **Distress level**^4^ |  |  |  |  |  |  |  |  |  |  |
| Mild | 129 | 42 | 32 | 47 | 41 | 39 | 35 | 48 | 19 | 34 |
| Clinically elevate | 177 | 58 | 35 | 53 | 68 | 1 | 37 | 52 | 36 | 66 |
| **Risk of malnutrition**^5^ |  |  |  |  |  |  |  |  |  |  |
| Yes | 97 | 30 | 15 | 21 | 33 | 29 | 24 | 30 | 25 | 43 |
| No | 223 | 70 | 55 | 79 | 79 | 71 | 56 | 70 | 33 | 57 |

^1^ Participants of survey study conducted in Verona, Italy, from February 2020 to May 2021. ^2^The number for each variable may differ due to missing values.  ^3^ Exercise level according to [1]. ^4^ Distress level according to [2]. ^5^ Risk of malnutrition according to [3].

1. Campbell, K.L., et al., *Exercise Guidelines for Cancer Survivors: Consensus Statement from International Multidisciplinary Roundtable.* Med Sci Sports Exerc, 2019. **51**(11): p. 2375-2390.

2. Riba, M.B., et al., *Distress Management, Version 3.2019, NCCN Clinical Practice Guidelines in Oncology.* J Natl Compr Canc Netw, 2019. **17**(10): p. 1229-1249.

3. Kondrup, J., et al., *Nutritional risk screening (NRS 2002): a new method based on an analysis of controlled clinical trials.* Clin Nutr, 2003. **22**(3): p. 321-36.

**Table 2.** Logistic modeling of associations of characteristics of patients with cancer with willingness to participate in a multimodal program^1^

|  | | **All (N)** | **OR^2^** | **95% CI^3^** | **P-value** |
| --- | --- | --- | --- | --- | --- |
| Age | <65 years (reference) | 184 | 1 |  |  |
|  | > 65 years | 140 | 0.42 | 0.26; 0.69 | < 0.001 |
| Sex | Female (reference) | 271 | 1 |  |  |
|  | Male | 183 | 0.75 | 0.47; 1.20 | 0.23 |
| BMI | < 25 kg/m2 (reference) | 178 | 1 |  |  |
|  | > 25 kg/m2 | 146 | 0,82 | 0.51; 1,30 | 0.39 |
| Education | Up to age 14 years (reference) | 128 | 1 |  |  |
|  | Beyond age 14 years | 196 | 1.48 | 0.88; 2.49 | 0.14 |
| Perceived income adequacy | Inadequate (reference) | 73 | 1 |  |  |
|  | Adequate | 251 | 0.74 | 0.42; 1.29 | 0.29 |
| Marital status | Married (reference) | 228 | 1 |  |  |
|  | Single | 35 | 2.24 | 1.08;4.63 | 0.03 |
|  | Divorced | 33 | 0.66 | 0.25; 1.74 | 0.40 |
|  | Widowed | 28 | 1.36 | 0.64;2.90 | 0.43 |
| Occupational status | Retired (reference) | 154 | 1 |  |  |
|  | Homemaker | 47 | 1.09 | 0.53; 2.24 | 0.82 |
|  | Part-time employed | 24 | 3.59 | 1.48; 8.72 | 0.005 |
|  | Full-time employed | 93 | 1.80 | 1.04; 3.12 | 0.03 |
| Distress | Mild level (reference) | 129 | 1 |  |  |
|  | Relevant level | 177 | 1.28 | 0.79; 2.08 | 0.31 |
| Aerobic activity | Inadequate (reference) | 220 | 1 |  |  |
|  | Adequate | 94 | 0.88 | 0.52; 1.46 | 0.61 |
| Strength activity | Inadequate (reference) | 283 | 1 |  |  |
|  | Adequate | 31 | 2.00 | 0.94; 4.27 | 0.07 |
| Aerobic + strength activity | Inadequate (reference) | 303 | 1 |  |  |
|  | Adequate | 11 | 0.66 | 0.20; 2.11 | 0.48 |
| Nutritional risk | No (reference) | 55 | 1 |  |  |
|  | Yes | 18 | 0.74 | 0.44; 1.23 | 0.24 |
| Tumor site | Breast (reference) | 20 | 1 |  |  |
|  | Lung | 143 | 0.49 | 0.15; 1.60 | 0.24 |
|  | Colorectal | 19 | 0.97 | 0.34; 1.76 | 0.95 |
|  | Upper gastro-intestine | 24 | 0.59 | 0.31; 1.11 | 0.10 |
|  | Head/neck | 22 | 0.54 | 0.18; 1.64 | 0.28 |
|  | Urogenital system | 23 | 0.47 | 0.17; 1.32 | 0.16 |
|  | Melanoma | 67 | 0.12 | 0.02; 0.57 | 0.007 |
|  | Other | 44 | 0.41 | 0.13; 1.32 | 0.14 |
| Disease status | Remission (reference) | 72 | 1 |  |  |
|  | Early | 82 | 0.34 | 0.14; 0.86 | 0.02 |
|  | Advanced | 59 | 0.99 | 0.49; 2.00 | 0.97 |
|  | Metastatic | 167 | 1.06 | 0.54; 2.08 | 0.78 |
|  | Unknown | 157 | 0.83 | 0.38; 1.81 | 0.65 |
| Surgery | No (reference) | 103 | 1 |  |  |
|  | Yes | 221 | 1.18 | 0.73; 1.91 | 0.50 |
| Chemotherapy | No (reference) | 251 | 1 |  |  |
|  | Yes | 73 | 1.00 | 0.62; 1.63 | 0.99 |
| Radiotherapy | No (reference) | 193 | 1 |  |  |
|  | Yes | 101 | 1.59 | 0.92; 2.74 | 0.09 |
| Hormone therapy | No (reference) | 193 | 1 |  |  |
|  | Yes | 101 | 1.36 | 0.56; 3.29 | 0.50 |
| Other | No (reference) | 43 | 1 |  |  |
|  | Yes | 16 | 1.03 | 0.52; 2.03 | 0.94 |
| Treatment status | Completed (reference) | 234 | 1 |  |  |
|  | About to start | 12 | 1.87 | 0.58; 5.98 | 0.29 |
|  | Ongoing | 186 | 1.05 | 0.53; 2.07 | 0.90 |
|  | Unknown | 138 | 0.62 | 0.15; 2.65 | 0.52 |
| Time from diagnosis | < 30 months (reference) | 184 | 1 |  |  |
|  | ≥ 30 months | 140 | 1.029 | 0.64; 1.66 | 0.91 |

^1^ willingness classified as yes vs. no/maybe; ^2^ OR (odds ratios); ^3^ CI (confidence intervals);

**Table 3.** Logistic modeling of associations of characteristics of patients with cancer with distress level^1^

|  | | **All (N)** | **OR^2^** | **95% CI^3^** | **P-value** |
| --- | --- | --- | --- | --- | --- |
| Age | <65 years (reference) | 184 | 1 |  |  |
|  | > 65 years | 140 | 0.98 | 0.62; 1.56 | 0.95 |
| Sex | Female (reference) | 271 | 1 |  |  |
|  | Male | 183 | 0.64 | 0.41; 1.01 | 0.06 |
| BMI | < 25 kg/m2 (reference) | 178 | 1 |  |  |
|  | > 25 kg/m2 | 146 | 1.12 | 0.71; 1.77 | 0.63 |
| Education | Up to age 14 years (reference) | 128 | 1 |  |  |
|  | Beyond age 14 years | 196 | 1.42 | 0.87; 2.33 | 0.16 |
| Perceived income adequacy | Inadequate (reference) | 73 | 1 |  |  |
|  | Adequate | 251 | 0.76 | 0.42; 1.34 | 0.34 |
| Marital status | Married (reference) | 228 | 1 |  |  |
|  | Single | 35 | 0.44 | 0.21; 0.92 | 0.03 |
|  | Divorced | 33 | 1.06 | 0.49; 2.27 | 0.88 |
|  | Widowed | 28 | 0.85 | 0.37; 1.97 | 0.71 |
| Occupational status | Retired (reference) | 154 | 1 |  |  |
|  | Homemaker | 47 | 1.45 | 0.73; 2.89 | 0.30 |
|  | Part-time employed | 24 | 0.63 | 0.27; 1.51 | 0.30 |
|  | Full-time employed | 93 | 0.89 | 0.52; 1.52 | 0.68 |
| Aerobic activity | Inadequate (reference) | 129 | 1 |  |  |
|  | Adequate | 177 | 0.50 | 0.31; 0.83 | 0.007 |
| Strength activity | Inadequate (reference) | 220 | 1 |  |  |
|  | Adequate | 94 | 0.47 | 0.21; 1.06 | 0.07 |
| Aerobic + strength activity | Inadequate (reference) | 283 | 1 |  |  |
|  | Adequate | 31 | 0.31 | 0.09; 1.03 | 0.06 |
| Nutritional risk | No (reference) | 303 | 1 |  |  |
|  | Yes | 11 | 2.82 | 1.65; 4.83 | <0.001 |
| Tumor site | Breast (reference) | 55 | 1 |  |  |
|  | Lung | 18 | 1.15 | 0.34; 3.89 | 0.83 |
|  | Colorectal | 20 | 0.80 | 0.27; 2.34 | 0.68 |
|  | Upper gastro-intestine | 143 | 0.83 | 0.43; 1.58 | 0.56 |
|  | Head/neck | 19 | 0.80 | 0.27; 2.34 | 0.68 |
|  | Urogenital system | 24 | 0.99 | 0.36; 2.69 | 0.98 |
|  | Melanoma | 22 | 0.88 | 0.30; 2.53 | 0.81 |
|  | Other | 23 | 0.71 | 0.25; 2.03 | 0.52 |
| Disease status | Remission (reference) | 67 | 1 |  |  |
|  | Early | 44 | 0.65 | 0.29; 1.45 | 0.29 |
|  | Advanced | 72 | 1.87 | 0.90; 3.86 | 0.09 |
|  | Metastatic | 82 | 1.29 | 0.65; 2.55 | 0.47 |
|  | Unknown | 59 | 0.91 | 0.42; 1.97 | 0.81 |
| Surgery | No (reference) | 167 | 1 |  |  |
|  | Yes | 157 | 1.11 | 0.70; 1.78 | 0.65 |
| Chemotherapy | No (reference) | 103 | 1 |  |  |
|  | Yes | 221 | 1.10 | 0.68; 1.75 | 0.72 |
| Radiotherapy | No (reference) | 251 | 1 |  |  |
|  | Yes | 73 | 1.27 | 0.73; 2.19 | 0.40 |
| Hormone therapy | No (reference) | 193 | 1 |  |  |
|  | Yes | 101 | 1.51 | 0.59; 3.87 | 0.39 |
| Other | No (reference) | 193 | 1 |  |  |
|  | Yes | 101 | 1.23 | 0.63; 2.41 | 0.55 |
| Treatment status | Completed (reference) | 43 | 1 |  |  |
|  | About to start | 16 | 1.31 | 0.38; 4.50 | 0.67 |
|  | Ongoing | 234 | 0.81 | 0.42; 1.58 | 0.54 |
|  | Unknown | 12 | 6.54 | 0.77; 55.83 | 0.09 |
| Time from diagnosis | < 30 months (reference) | 186 | 1 |  |  |
|  | ≥ 30 months | 138 | 0.75 | 0.47; 1.20 | 0.23 |

^1^ distress classified as > 3 vs. < 4; ^2^ OR (odds ratios); ^3^ CI (confidence intervals);

**Table 4.** Logistic modeling of associations of characteristics of patients with cancer with aerobic exercise level^1^

|  | | **All (N)** | **OR^2^** | **95% CI^3^** | **P-value** |
| --- | --- | --- | --- | --- | --- |
| Age | <65 years (reference) | 184 | 1 |  |  |
|  | > 65 years | 140 | 1.01 | 0.62; 1.65 | 0.96 |
| Sex | Female (reference) | 271 | 1 |  |  |
|  | Male | 183 | 1.38 | 0.85; 2.24 | 0.19 |
| BMI | < 25 kg/m2 (reference) | 178 | 1 |  |  |
|  | > 25 kg/m2 | 146 | 0.93 | 0.57; 1.51 | 0.77 |
| Education | Up to age 14 years (reference) | 128 | 1 |  |  |
|  | Beyond age 14 years | 196 | 1.08 | 0.64; 1.82 | 0.78 |
| Perceived income adequacy | Inadequate (reference) | 73 | 1 |  |  |
|  | Adequate | 251 | 2.18 | 1.10; 4.31 | 0.03 |
| Marital status | Married (reference) | 228 | 1 |  |  |
|  | Single | 35 | 0.97 | 0.45; 2.10 | 0.94 |
|  | Divorced | 33 | 0.51 | 0.18; 1.41 | 0.19 |
|  | Widowed | 28 | 0.31 | 0.11; 0.93 | 0.04 |
| Occupational status | Retired (reference) | 154 | 1 |  |  |
|  | Homemaker | 47 | 0.59 | 0.27; 1.28 | 0.18 |
|  | Part-time employed | 24 | 1.16 | 0.46; 2.93 | 0.75 |
|  | Full-time employed | 93 | 0.95 | 0.54; 1.67 | 0.86 |
| Distress | Mild (reference) | 129 | 1 |  |  |
|  | Clinically relevant | 177 | 0.50 | 0.31; 0.83 | 0.007 |
| Strength activity | Inadequate (reference) | 220 | 1 |  |  |
|  | Adequate | 94 | 2.81 | 1.30; 6.10 | 0.009 |
| Nutritional risk | No (reference) | 283 | 1 |  |  |
|  | Yes | 31 | 0.80 | 0.47; 1.37 | 0.42 |
| Tumor site | Breast (reference) | 303 | 1 |  |  |
|  | Lung | 11 | 1.13 | 0.33; 3.83 | 0.85 |
|  | Colorectal | 55 | 0.80 | 0.25; 2.61 | 0.72 |
|  | Upper gastro-intestine | 18 | 0.99 | 0.50; 1.98 | 0.99 |
|  | Head/neck | 20 | 1.80 | 0.60; 5.41 | 0.30 |
|  | Urogenital system | 143 | 0.79 | 0.26; 2.39 | 0.68 |
|  | Melanoma | 19 | 0.96 | 0.31; 2.97 | 0.95 |
|  | Other | 24 | 0.80 | 0.25; 2.61 | 0.72 |
| Disease status | Remission (reference) | 22 | 1 |  |  |
|  | Early | 23 | 0.88 | 0.38; 2.04 | 0.77 |
|  | Advanced | 67 | 0.61 | 0.28; 1.33 | 0.21 |
|  | Metastatic | 44 | 1.07 | 0.53; 2.18 | 0.85 |
|  | Unknown | 72 | 1.12 | 0.50; 2.51 | 0.78 |
| Surgery | No (reference) | 82 | 1 |  |  |
|  | Yes | 59 | 0.93 | 0.56; 1.53 | 0.77 |
| Chemotherapy | No (reference) | 167 | 1 |  |  |
|  | Yes | 157 | 0.71 | 0.43; 1.19 | 0.19 |
| Radiotherapy | No (reference) | 103 | 1 |  |  |
|  | Yes | 221 | 1.44 | 0.82; 2.54 | 0.21 |
| Hormone therapy | No (reference) | 251 | 1 |  |  |
|  | Yes | 73 | 1.91 | 0.76; 4.78 | 0.17 |
| Other | No (reference) | 193 | 1 |  |  |
|  | Yes | 101 | 1.71 | 0.87; 3.37 | 0.12 |
| Treatment status | Completed (reference) | 193 | 1 |  |  |
|  | About to start | 101 | 0.43 | 0.11; 1.75 | 0.24 |
|  | Ongoing | 43 | 0.86 | 0.43; 1.70 | 0.66 |
|  | Unknown | 16 | 0.19 | 0.02; 1.60 | 0.13 |
| Time from diagnosis | < 30 months (reference) | 234 | 1 |  |  |
|  | ≥ 30 months | 12 | 2.41 | 1.46; 3.98 | <0.001 |
|  |  | 186 |  |  |  |
|  |  | 138 |  |  |  |

^1^ aerobic exercise classified as > 89 min/week vs. < 90 min/week; ^2^ OR (odds ratios); ^3^ CI (confidence intervals);

**Table 4.** Logistic modeling of associations of characteristics of patients with cancer with nutritional risk^1^

|  | | **All (N)** | **OR^2^** | **95% CI^3^** | **P-value** |
| --- | --- | --- | --- | --- | --- |
| Age | <65 years (reference) | 184 | 1 |  |  |
|  | > 65 years | 140 | 1.57 | 0.97; 2.53 | 0.07 |
| Sex | Female (reference) | 271 | 1 |  |  |
|  | Male | 183 | 0.67 | 0.41; 1.08 | 0.10 |
| BMI | < 25 kg/m2 (reference) | 178 | 1 |  |  |
|  | > 25 kg/m2 | 146 | 0.28 | 0.17; 0.46 | <0.001 |
| Education | Up to age 14 years (reference) | 128 | 1 |  |  |
|  | Beyond age 14 years | 196 | 1.09 | 0.65; 1.83 | 0.75 |
| Perceived income adequacy | Inadequate (reference) | 73 | 1 |  |  |
|  | Adequate | 251 | 1.69 | 0.90; 3.19 | 0.10 |
| Marital status | Married (reference) | 228 | 1 |  |  |
|  | Single | 35 | 0.86 | 0.38; 1.94 | 0.72 |
|  | Divorced | 33 | 0.89 | 0.36; 2.23 | 0.80 |
|  | Widowed | 28 | 1.04 | 0.47; 2.32 | 0.92 |
| Occupational status | Retired (reference) | 154 | 1 |  |  |
|  | Homemaker | 47 | 0.88 | 0.44; 1.77 | 0.72 |
|  | Part-time employed | 24 | 0.48 | 0.17; 1.35 | 0.16 |
|  | Full-time employed | 93 | 0.54 | 0.30; 0.97 | 0.04 |
| Distress | Mild level (reference) | 129 | 1 |  |  |
|  | Relevant level | 177 | 2.82 | 1.65; 4.83 | <0.001 |
| Aerobic activity | Inadequate (reference) | 220 | 1 |  |  |
|  | Adequate | 94 | 0.80 | 0.47; 1.37 | 0.42 |
| Strength activity | Inadequate (reference) | 283 | 1 |  |  |
|  | Adequate | 31 | 0.63 | 0.26; 1.51 | 0.30 |
| Aerobic + strength activity | Inadequate (reference) | 303 | 1 |  |  |
|  | Adequate | 11 | 0.81 | 0.25; 2.60 | 0.72 |
| Tumor site | Breast (reference) | 55 | 1 |  |  |
|  | Lung | 18 | 1.17 | 0.32; 4.28 | 0.82 |
|  | Colorectal | 20 | 2.55 | 0.84; 7.76 | 0.10 |
|  | Upper gastro-intestine | 143 | 2.10 | 1.01; 4.33 | 0.04 |
|  | Head/neck | 19 | 1.00 | 0.28; 3.61 | 1.00 |
|  | Urogenital system | 24 | 1.24 | 0.40; 3.83 | 0.71 |
|  | Melanoma | 22 | 0.66 | 0.16;2.64 | 0.55 |
|  | Other | 23 | 1.25 | 0.37; 4.18 | 0.72 |
| Disease status | Remission (reference) | 67 | 1 |  |  |
|  | Early | 44 | 2.71 | 1.16; 6.33 | 0.02 |
|  | Advanced | 72 | 2.71 | 1.26; 5.81 | 0.01 |
|  | Metastatic | 82 | 0.94 | 0.42; 2.10 | 0.88 |
|  | Unknown | 59 | 1.31 | 0.55; 3.14 | 0.54 |
| Surgery | No (reference) | 167 | 1 |  |  |
|  | Yes | 157 | 0.69 | 0.42; 1.13 | 0.14 |
| Chemotherapy | No (reference) | 103 | 1 |  |  |
|  | Yes | 221 | 1.31 | 0.79; 2.15 | 0.29 |
| Radiotherapy | No (reference) | 251 | 1 |  |  |
|  | Yes | 73 | 0.73 | 0.41; 1.33 | 0.31 |
| Hormone therapy | No (reference) | 193 | 1 |  |  |
|  | Yes | 101 | 1.31 | 0.53; 3.24 | 0.56 |
| Other | No (reference) | 193 | 1 |  |  |
|  | Yes | 101 | 0.85 | 0.41; 1.74 | 0.65 |
| Treatment status | Completed (reference) | 43 | 1 |  |  |
|  | About to start | 16 | 6.43 | 1.79; 23.08 | 0.004 |
|  | Ongoing | 234 | 2.34 | 0.99; 5.52 | 0.05 |
|  | Unknown | 12 | 2.50 | 0.59; 10.65 | 0.22 |
| Time from diagnosis | < 30 months (reference) | 186 | 1 |  |  |
|  | ≥ 30 months | 138 | 0.73 | 0.44; 1.20 | 0.22 |

^1^ nutritional risk classified as > 2 vs. < 3; ^2^ OR (odds ratios); ^3^ CI (confidence intervals);
